# Serum Total Thyroxine Evaluation in Critically Ill Feline Patients

**DOI:** 10.3390/vetsci8020021

**Published:** 2021-01-27

**Authors:** Eleonora Gori, Alessio Pierini, Erica Bartolomeo, Gianila Ceccherini, Anna Pasquini, Veronica Marchetti

**Affiliations:** Veterinary Teaching Hospital “Mario Modenato”, Department of Veterinary Sciences, University of Pisa, Via Livornese Lato monte, San Piero a Grado, 56122 Pisa, Italy; Eleonora.gori@vet.unipi.it (E.G.); erica.bartolomeo@virgilio.it (E.B.); gianilaceccherini@virgilio.it (G.C.); anna.pasquini@unipi.it (A.P.); veronica.marchetti@unipi.it (V.M.)

**Keywords:** thyroid, intensive care, prognosis, non-thyroidal illness syndrome

## Abstract

This retrospective case control study compared serum total thyroxine (tT4) concentrations in hospitalized critical cats (CCs) and non-hospitalized cats with non-thyroidal chronic diseases (chronic group, CG) and evaluated the relationship between the serum tT4 concentration of CCs and systemic inflammation (systemic inflammatory response syndrome (SIRS)), disease severity (Acute Patient Physiologic and Laboratory Evaluation (APPLE_fast_)), and prognosis. Cats with previously suspected or diagnosed thyroid disease were excluded. Serum tT4 was evaluated in surplus serum samples at the time of admission for CCs and CGs. The APPLE_fast_ score of the CC group was calculated at admission. The systemic inflammatory response syndrome (SIRS) in CCs was determined using proposed criteria. Cats were divided into survivors and non-survivors according to the discharge outcome. Forty-nine cats were retrospectively included. Twenty-seven cats died during hospitalization. The CG group was composed of 37 cats. The CC group showed a significantly lower tT4 compared to the CG group (1.3 ± 0.7 vs. 2 ± 0.9; *p* < 0.0001). Among SIRS, APPLE_fast_, and tT4, only tT4 was associated with mortality (*p* = 0.04). The tT4 cut-off point for mortality was 1.65 μg/dL (sensitivity 81%, specificity 57%, odds ratio (OR) 5.6). Twenty-five cats (51%) had SIRS that was not associated with tT4. Non-thyroidal illness syndrome can occur in critically ill cats and the evaluation of tT4 in hospitalized cats could add prognostic information.

## 1. Introduction

During critical illnesses in humans, serum thyroid hormone levels are known to decrease [1,2,3,4]. In mild diseases, this only leads to a decrease in serum triiodothyronine (T3) levels. However, with increasing disease severity and duration, both serum T3 and thyroxine (T4) levels may be decreased [1,5]. This pathological state has been defined as non-thyroidal illness syndrome (NTIS) [1,5]. NTIS is characterized by a decreased serum concentration and reduced tissue supply of thyroid hormones, derived from a combination of hypothyroidism and altered peripheral metabolism of T4 and T3 [1,5]. According to a two-year period study, 23% of ICU human patients with low thyroid hormone levels (T3 and T4) and normal or low TSH have an increased risk of death [6]. 

Despite numerous studies in human medicine, NTIS has not been extensively studied in veterinary medicine. Some studies conducted on dogs showed significantly lower thyroid hormone levels in critically ill patients [7,8,9]. In addition, lower thyroid hormone concentrations were associated with disease severity and a systemic inflammatory response syndrome (SIRS) [7,9]. Low T3 and T4 concentrations and the simultaneous presence of SIRS, can be considered as an expression of NTIS in critically ill canine patients [9]. 

To the best of our knowledge, NTIS in cats has only been evaluated in three studies [10,11,12], and in a conference abstract [13]. In these three studies [10,11,12], the total T4 (tT4) concentration was significantly lower in ill cats than in healthy cats. In addition, tT4 was related to the disease severity and was significantly lower in non-survivor cats than survivors, therefore attributing a possible prognostic value to the decrease in tT4 in these patients [10,11,12]. Davignon et al. showed that the median serum tT4 and fT4 were significantly lower in 46 ill cats compared to the healthy ones [13]. Peterson et al. recently reported findings showing that tT4 was different both in relation to the presence of an NTIS and mortality; however, both fT4 and TSH did not differ from healthy compared to NTIS cats. [14]. 

We hypothesized that, as in humans and dogs, non-survivor critical cats and cats with more severe diseases may have lower total T4 levels than survivor cats with mild diseases. Data about study was presented as an abstract (<250 words) and a poster presentation at the EVECC Congress in Tallinn, Estonia, 2019 [15]. The aims of our study were (1) to investigate the role of serum tT4 concentrations as a prognostic indicator in critically ill feline patients and to compare tT4 with non-hospitalized cats with non-thyroidal chronic disease, and (2) to evaluate the relationship between the serum tT4 concentration in critical cats and the presence of systemic inflammation (SIRS) and Acute Patient Physiologic and Laboratory Evaluation (APPLE_fast_).

## 2. Materials and Methods 

A retrospective search of medical records was conducted in our veterinary teaching hospital’s database with regards to cats that were hospitalized (critical cats, CCs) from July 2014 to July 2018. Admission records were reviewed by one clinician (E.B.) to identify cats that had been hospitalized in the intensive care unit under red (immediate assistance), orange (very urgent assistance, 15 min waiting), or yellow (urgent assistance, maximum 30–60 min waiting) codes [16]. Cats previously suspected or diagnosed with thyroid diseases, cats with incomplete medical records, or cats for which serum and hematological profiles were not available at admission, were excluded. 

Excess serum samples of animals admitted to our hospital are routinely stored at −80 °C. With the owner’s informed consent, tT4 of CC was measured in surplus serum samples stored at the time of hospital admission (T0) using a competitive fluorescent enzyme immunoassay system (AIA-360, Tosoh Bioscience, Tokyo, Japan). The AIA-360 had been previously validated for feline tT4 evaluation [17]. Mean within and between-run coefficients of variation for T4 were ≤7% (range 3.3–11.2%), whereas lower and higher limits of detection were 0.5 and 24 μg/dL, respectively [17]. 

Concentrations of tT4 in small animal serum samples were stable from 3 months to 2 years when stored at −20 °C [9,17,18,19,20], while human serum tT4 demonstrated a stability for almost 7 years at −80 °C [21]. Our serum samples were stored from a minimum of 3 months to a maximum of 4 years at −80 °C. In addition, a retrospective review of medical records was conducted to search for non-hospitalized cats with non-thyroidal chronic diseases (chronic group, CG) admitted to our hospital in the same study period, for which the tT4 was available. 

The presence of SIRS was retrospectively evaluated on the basis of medical records. Cats were diagnosed with SIRS if they showed at least 2 of the following criteria: (1) abnormal temperature (≤37.8 or ≥39.7 °C), (2) tachycardia (≥225 beats/min) or bradycardia (≤140 beats/min), (3) tachypnea (≥40 breaths/min), and (4) WBC abnormalities (WBC ≥ 19,500 or ≤5000 k/μL or band neutrophils ≥5%) [22]. 

In each cat, the feline Acute Patient Physiologic and Laboratory Evaluation (APPLE_fast_) score [23] was retrospectively calculated on the basis of the parameters of the medical records at hospital admission. The APPLE_fast_ score is a validated, user-friendly score to stratify illness severity in hospitalized cats. It is a 5-variable score that contains an evaluation of the mentation score, temperature, mean arterial pressure, lactate, and PCV [23]. The total score is calculated by summing the values for each alteration of the 5 parameters listed above (maximum score of 50) [23]. Cats without some clinical or hematological parameters for the SIRS and APPLE_fast_ calculations were excluded.

Cats were also divided into survivors and non-survivors according to the discharge outcome, which was extrapolated from medical records: survivors (discharged from the hospital) and non-survivors (died during hospitalization). The underlying disease category and the reason for death (spontaneous or euthanasia) were also recorded.

A Kolmogorov–Smirnov test was used to assess the continuous variables distribution (age, tT4, APPLE_fast_). Serum tT4 was compared between groups (CC vs. CG and SIRS vs. non-SIRS) using the unpaired *t*-test since tT4 was normally distributed in these groups. In addition, tT4 was correlated with APPLE_fast_ using Pearson’s correlation test. A multivariable backward stepwise binary logistic regression was performed to assess the association of tT4 and presence of SIRS and APPLE_fast_ with mortality. If a variable remained significantly associated with mortality (*p* < 0.05), it was considered to be associated with the mortality. A receiver operating characteristic (ROC) curve was built in order to find the better cut-off value of tT4 for mortality. For tT4, a ROC analysis was performed and the area under the curve (AUC) was calculated. The odds ratio (OR) was then calculated. The diagnostic cut-off and its sensitivity and specificity were determined according to the maximum Youden index. Statistical analysis was performed using IBM SPSS Statistics v.25 (IBM Corporation, New York, NY, USA) and a *p*-value < 0.05 was considered statistically significant.

## 3. Results

The CC group was composed of 49 cats. The mean age was 6.9 ± 3 years. Of the cats, 23 were female, 16 of which were spayed, and 26 were male, 19 of which were neutered. The majority of CCs were Domestic Shorthair (38/49), and of the remaining cats, five were Persian, three were Birman, one was Ragdoll, one was Siberian, and one was Exotic Shorthair. In the CCs, the reasons for hospitalization were nephrourological diseases (*n* = 18), gastrointestinal diseases (*n* = 13), trauma (*n* = 7), hematological diseases (*n* = 4), infectious and cardiovascular diseases (*n* = 3 each), and one respiratory disease.

The CG group was composed of 37 cats. The mean age of the CG group was 12.5 ± 3 years, which was significantly higher than the CC group (*p* < 0.0001). A total of 14 cats were female, 11 of which were spayed, and 23 were male, 21 of which were neutered. In the CG group there were 16 cats with gastrointestinal diseases, 13 cats with nephrourological diseases, 4 cats with neoplastic diseases, 3 with respiratory diseases, and 1 cat with diabetes mellitus. 

The mean tT4 was significantly lower in CC than in CG (1.3 ± 0.7 vs. 2 ± 0.9 μg/dL; *p* < 0.0001). On the basis of the criteria used for the diagnosis of SIRS, 25 out of 49 CC (51%) had SIRS. There was no statistically significant difference in mean tT4 between cats with or without SIRS (1.3 ± 0.8 vs. 1.4 ± 0.7; *p* = 0.62). The APPLE_fast_ of the CC group ranged from 10 to 35 points. There was no correlation between the APPLE_fast_ score and tT4 (*p* = 0.87). 

Lastly, evaluating the mortality of CC, in this study, 27 cats (55.1%) belonged to the non-survivors and the remaining 22 belonged to the survivors. Out of the 27 cats, 7 underwent euthanasia due to a progressive deterioration of the clinical condition despite treatment. No cat underwent euthanasia due to financial concerns. 

In the binary logistic regression analysis including tT4, and in the presence of SIRS and APPLE_fast_, only tT4 was associated with mortality (*p* = 0.04; Table 1). The optimal cut-off point for tT4 for mortality was 1.65 μg/dL (sensitivity 81%, specificity 57%, AUC 0.7 95% CI 0.5–0.82, OR 5.1 95% CI 1.5–17.5).

## 4. Discussion

We investigated serum tT4 concentrations in critically ill hospitalized cats (CCs), comparing them to a group of non-hospitalized cats with non-thyroidal chronic diseases, finding that tT4 may have a possible prognostic value in such cats. 

The hypothesis behind the present study stemmed from human and small animal studies [5,6,7,8,9,10,11,12,13,14,24,25] that have demonstrated a thyroid hormone alteration in critically ill euthyroid patients. The concept of NTIS is used in both veterinary and human medicine. In the initial stages, NTIS seems to be an adaptive mechanism to reduce energy expenditure [26], while in the prolonged and more severe disease phase, it appears to be a maladaptive response with a decrease in thyroid hormones and other pituitary hormones [27]. We focused on tT4 because it is very widely available and easily measurable by clinicians in routine inhouse laboratory testing, whereas T3 and TSH are more expensive and difficult to assess. 

We used a control group composed of non-hospitalized cats with chronic non-thyroidal illnesses, instead of a group of healthy cats, in order to better study the role of serum tT4 in critically ill cats. In fact, a control group of cats with chronic non-thyroidal illnesses enabled us to better study the behavior of the thyroid axis in critically ill cats compared to other and non-hospitalization-required sicknesses, since there are already sufficient data regarding thyroid hormones and NTIS in cats [10,11,12,13,14].

Our CC population was primarily composed of younger cats compared to the CG group (non-hospitalized cats with non-thyroidal illnesses). This may be easily explained by the nature of most chronic diseases, which are persistent over a significant time period before causing death, or may not be a cause of death, and are therefore more common in older cats. Also, younger cats are not routinely screened for hyperthyroidism in contrast to elderly cats

In line with the current human and feline veterinary literature [10,11,12,13,14], our results showed that in cats with critical non-thyroid diseases (CC), the serum tT4 concentration is lower than in non-hospitalized cats with chronic diseases. Alterations in circulating thyroid hormones are widely documented in human medicine and may affect about 60–70% of critically ill patients with various diseases [1]. 

NTIS is characterized by a decrease in the serum tT3 concentration and a simultaneous increase in serum reverse-T3 levels, together with low serum tT4; fT4; and, occasionally, TSH [1]. There are three main hypothesized mechanisms regarding the pathogenesis of NTIS. Firstly, the presence of thyroid-binding hormone inhibitor in serum and body tissues may inhibit the binding of thyroid hormones to thyroid-binding proteins [1,24,27,28]. Secondly, NTIS may also be caused by inflammatory cytokines that can affect the hypothalamus–pituitary axis, inhibiting TSH, thyroid-releasing hormone, thyroglobulin, T3, and thyroid-binding globulin production [1,24,27,28]. Lastly, deiodinase type 3, which is the major TH inactivating enzyme, may play an important role [1,24,27,28]. The deiodinase type 3 catalyzes the deiodination of both T4 and T3, resulting in the production of biologically inactive rT3 [1,24,27,28].

Currently, the only study that has investigated the association between tT4 and SIRS in veterinary medicine was conducted by Giunti et al. [9]. The authors studied a canine population with NTIS, concluding that the serum tT4 concentration was significantly associated with the presence of SIRS. On the other hand, our hypothesis was that, although the SIRS criteria in cats have been questioned over the last few years, they are easily established, reproducible, and validated criteria for evaluating the presence of a systemic inflammatory process [22]. However, we failed to find an association between tT4 and SIRS. This could be due to the SIRS criteria in cats, which have been established in a population of cats that died from severe sepsis [22] and, therefore, may not be representative of all critical cats. 

Unlike findings reported in dogs [23], we found no significant association between the serum tT4 value and the clinical severity (APPLE_fast_ score). The APPLE score, in both the full and fast version, is the only currently validated score for assessing survival in hospitalized cats and provides a reproductible way of assessing disease severity.

Our results appear to be in line with all the other studies applying APPLE scales in cats and correlating them with mortality [29,30,31]. This finding could be due to the unclear assignment of APPLE scores to the variable values taken into consideration. In fact, APPLE scores have been ineffective in predicting mortality [31] outside the original study population [23], and thus their validity in other contexts remains unclear. Peterson et al. [14] tried to evaluate disease and NTIS severity using a clinician-based assessment, dividing cats on the basis of the need for hospitalization/ICU admission [14]. On the basis of their data, cats with mild NTIS (outpatients equivalent to our CGs) had tT4 within ranges but higher than severe NTIS cats (equivalent to our CCs), which is in line with our results.

We assessed the serum tT4 concentration in relation to mortality and found that non-survivors had a significantly lower tT4 than survivors. In accordance with human [5,6] and other veterinary studies [7,8,9,10,11,12,13], low tT4 serum concentration of may be indicative of NTIS and may be associated with a high probability of death. From the results of the ROC analysis, we identified 1.65 μg/dL as the best tT4 cutoff value for mortality. Briefly, approximately 80% of non-survivors had a tT4 < 1.65 μg/dL, whereas approximately 57% of survivors had a tT4 > 1.65 μg/dL. In addition, on the basis of the OR, CCs with a tT4 < 1.65 μg/dL showed a 5.6 times greater risk of death than patients with tT4 > 1.65 μg/dL. However, due to the low value of specificity, these data should be interpreted with caution.

This study had several limitations. Firstly, as feline patients may often be aggressive or uncooperative, or unsuitable for sedation due to the critical conditions, we were unable to use a larger population study. Moreover, we only evaluated tT4 at admission, although it might be interesting to monitor it in relation to the final outcome. Another limitation, due to the retrospective nature of the study, is the lack of TSH evaluation, which in combination with tT4 might add some prognostic information on NTIS cats. However, in Peterson’s study, TSH did not differ between healthy and NTIS cats and remained in the reference range in the majority of cases [14]. 

Not all of the samples had the same storage time. Consequently, since we only had data on long-term human and canine tT4 stability at −80 °C [9,16,17,18,19], we cannot rule out that a storage time longer than two years (maximum time for known stability of tT4 for small animals) [9] might affect the tT4 measurement. Furthermore, SIRS and APPLE_fast_ may not be the best means to score a critically ill cat, although, to date, there are no other specific scoring systems. Lastly, it would be interesting to evaluate tT4 in relation to various systems or apparatus diseases to find if there is a group of diseases that causes more severe NTIS than others.

## 5. Conclusions

Our results support the presence of a NTIS in critically ill hospitalized cats as well as the association between serum tT4 concentration and mortality. We believe that serum tT4 may be an additional prognostic marker in critically ill cats. Our results could open the way to new studies focused on evaluating the therapeutic use of thyroid hormones in critically ill cats, as happens in human medicine. 

## Figures and Tables

**Table 1:** Results of the multivariable backward stepwise binary logistic regression model for mortality including presence of systemic inflammatory response syndrome (SIRS), Acute Patient Physiologic and Laboratory Evaluation (APPLE_fast_), and total thyroxine (tT4).

		B	S.E.	Wald	dF	Sig.	OR	Lower 95% CI	Upper 95% CI
Phase 1	tT4	0.89	0.44	4.07	1	0.04	2.44	1.03	5.8
	SIRS	0.26	0.62	0.18	1	0.68	1.3	0.39	4.3
	APPLE_fast_	−0.03	0.05	0.43	1	0.51	0.97	0.88	1.03
Phase 2	tT4	0.91	0.44	4.17	1	0.04	2.47	1.04	5.9
	APPLE_fast_	−0.03	0.05	0.56	1	0.45	0.96	0.88	1.06
Phase 3	tT4	0.89	0.43	4.18	1	0.04	2.43	1.038	5.73

B, B statistic; S.E., standard error; Wald, Wald statistic; dF, degrees of freedom; Sig., significance; OR, odds ratio.

## Data Availability

The data presented in this study are available on reasonable request from the corresponding author. The data are not publicly available due to other project involvement.
